# Supplementary material for: Tissue-specific TCF4 triplet repeat instability revealed by optical genome mapping
Source: eBioMedicine. 2024 Sep 14;108:105328. doi: 10.1016/j.ebiom.2024.105328 (PMC11419830; doi:10.1016/j.ebiom.2024.105328)
Supplement: Supplementary Tables and Figures [file mmc2.docx]

# Supplemental Data


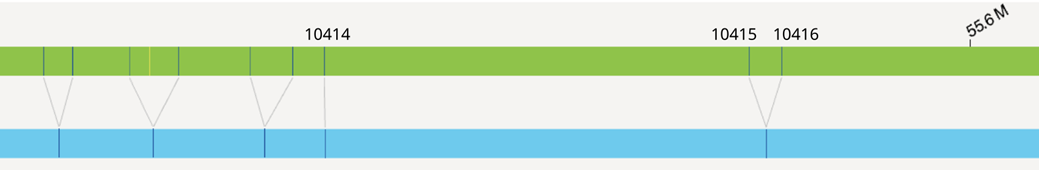


**Supplemental Figure S1:** A normal allele (blue) compared with the hg38 reference (green), with matching markers. Several markers, including marker of interest 10415, have more markers in close proximity (<1 kb), however only one of these markers will be detected.

**
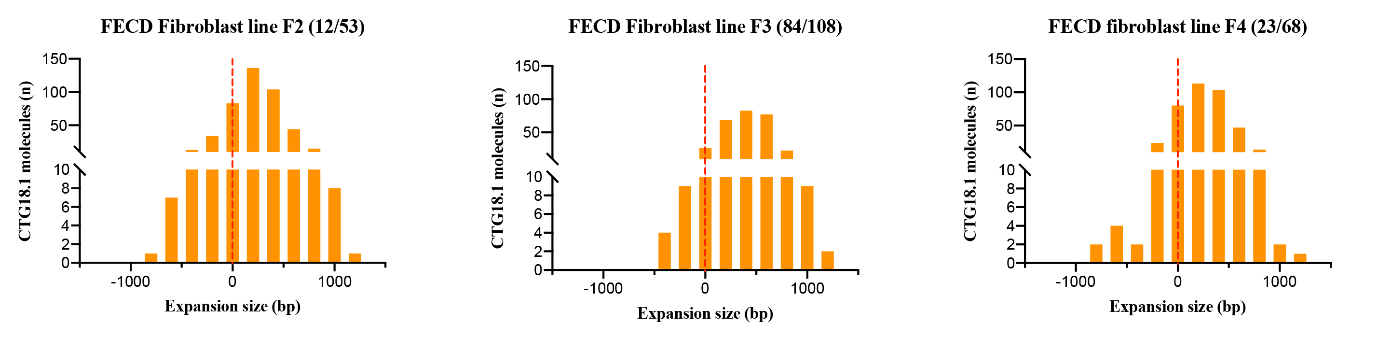
**

**Supplemental Figure S2: CTG18.1 is somatically stable in expansion-positive fibroblasts.** DNA was extracted from cultured dermal fibroblasts isolated from three CTG18.1 expansion-positive individuals with FECD and analysed by optical genome mapping. The size (bp) of the CTG18.1 repeat-containing molecules is plotted (x-axis) against the total number of CTG18.1 molecules detected (y-axis). Baseline CTG18.1 genotypes determined by STR-PCR are shown in brackets for each sample.


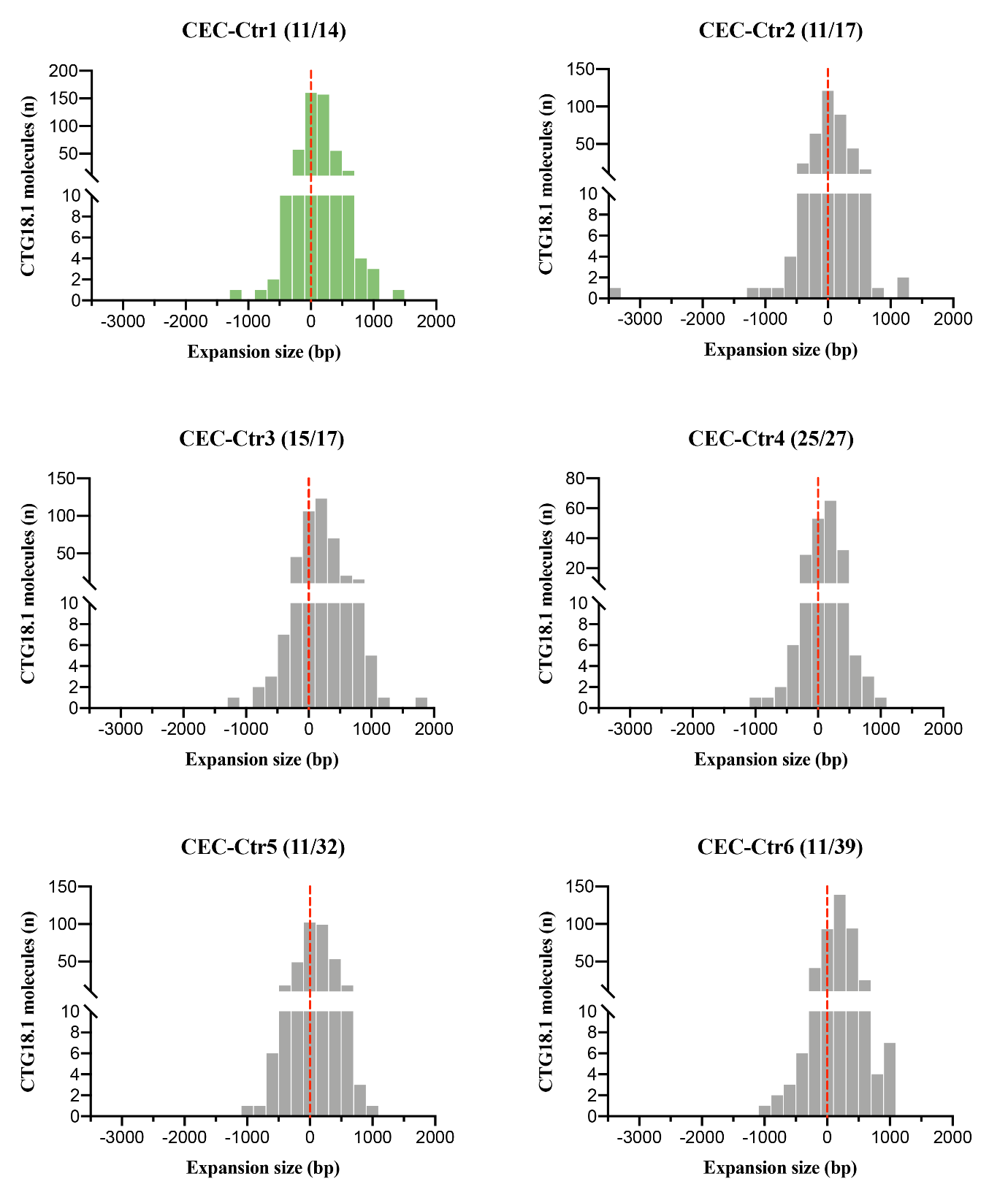


**Supplemental Figure S3: CTG18.1 is somatically stable in control corneal endothelial cells (CECs).** DNA was extracted from CECs derived from five healthy control lines (grey) and one CTG18.1 expansion-negative FECD-patient derived line (green) and analysed by optical genome mapping (CEC-Ctr1-6). The size (bp) of the CTG18.1 repeat-containing molecules is plotted (x-axis) against the total number of CTG18.1 molecules detected (y-axis). Baseline CTG18.1 genotypes determined by STR-PCR shown in brackets for each sample.


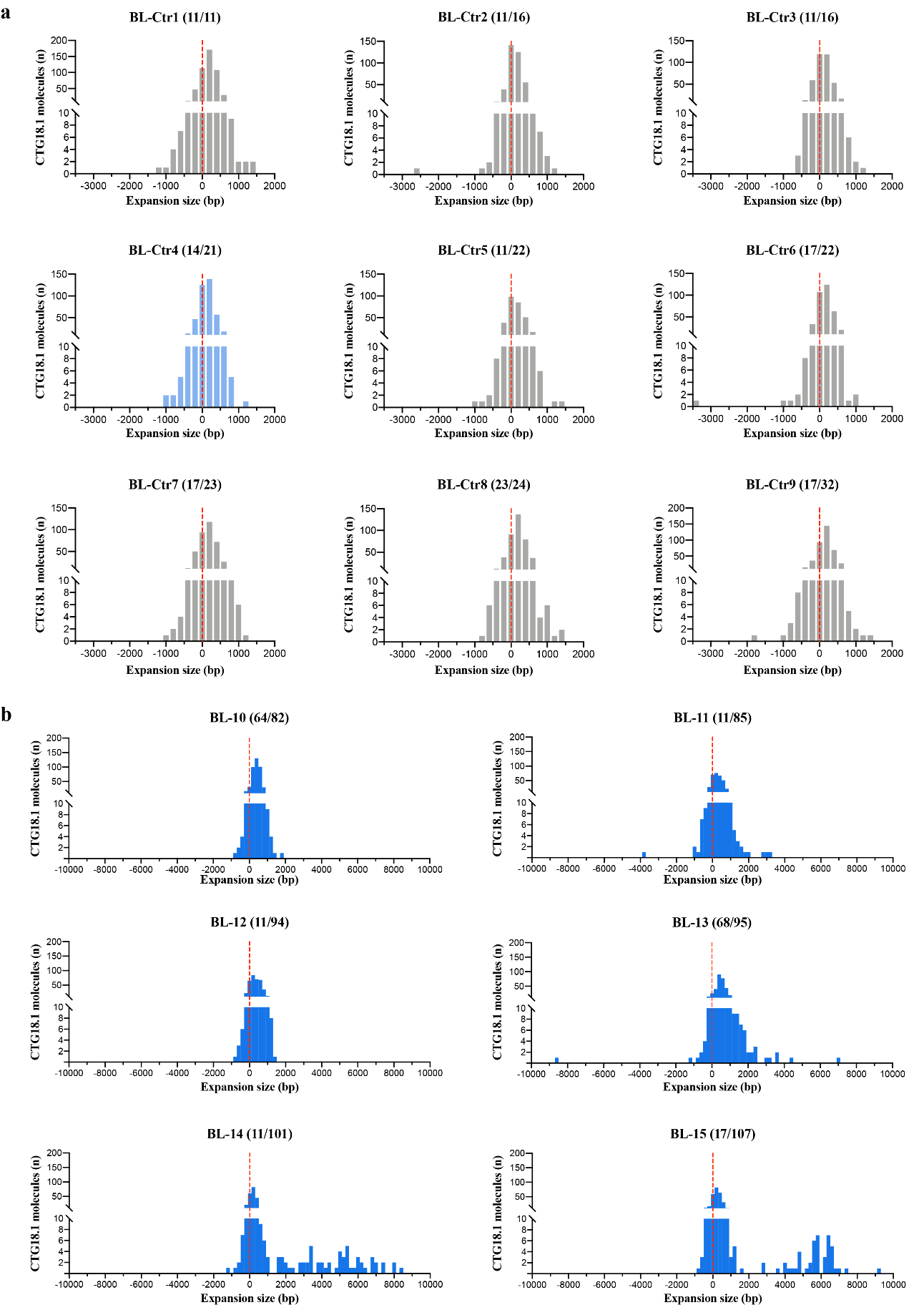


**Supplemental Figure S4: Exploring CTG18.1 instability across a subset of peripheral blood leukocyte samples with a diverse range of repeat sizes.** A series of peripheral blood leukocyte-derived (BL) gDNA samples from fifteen unrelated individuals were analysed by OGM. **(a)** Samples BL-Ctr1-9 were derived from eight unaffected (grey) or FECD (light blue) CTG18.1 expansion-negative individuals. **(b)** Samples BL-10-15 were derived from FECD individuals with CTG18.1 expansions. The size (bp) of the CTG18.1 repeat-containing molecules is plotted (x-axis) against the total number of CTG18.1 molecules detected (y-axis). Baseline CTG18.1 genotypes determined by STR-PCR analysis of leukocyte gDNA are shown in brackets.

| **Subject** | **Sex** | **FECD**  **Diagnosis** | **STR genotype** | **Subject sampling age** | **CUG RNA foci** | ***TCF4* isoform shift** | **OGM Mean allele length**  **(bp)** | **OGM Max allele length (bp)** | **Number of**  **CTG18.1 molecules** |
| --- | --- | --- | --- | --- | --- | --- | --- | --- | --- |
| #F2 | Female | ✓ | 12/53 | 71 | No^a^ | No^b^ | 224 | 1,127 | 446 |
| #F3 | Female | ✓ | 84/108 | 65 | No^a^ | No^b^ | 396 | 1,225 | 303 |
| #F4 | Female | ✓ | 23/68 | 78 | No^a^ | No^b^ | 249 | 1,129 | 392 |

**Table S1:** **Optical genome mapping molecule summary of human dermal fibroblasts isolated from CTG18.1 expansion-positive individuals with FECD.** a: Fibroblast isolation and CUG RNA foci data presented in Zarouchlioti et al. 2018. b: *TCF4* isoform RNAScope data presented in Bhattacharyya et al. 2024.

| **Spearman Correlation** | | |
| --- | --- | --- |
|  | **Blood** | **CEC** |
| All Data: Correlation (95% CI, *p*-value) | 0.255 (0.236-0.274, <0.0001) | 0.229 (0.201-0.256, <0.0001) |
| Expansion-positive: Correlation (95% CI, *p*-value) | 0.142 (0.117-0.168, <0.0001) | 0.071 (0.032-0.110, 0.0003) |
| Expansion-negative: Correlation (95% CI, *p*-value) | 0.037 (0.004-0.070, 0.026) | 0.085 (0.043-0.126, <0.0001) |

**Table S2:** Spearman correlation coefficient analysis exploring the relationship between the largest progenitor CTG18.1 allele and the total molecule sizes measured by OGM. Cases with ≥50 CTG repeats (STR analysis of leukocyte-derived DNA) on at least one allele were classified as expansion-positive.

| **Linear Regression Modelling Results** | | | | |
| --- | --- | --- | --- | --- |
| Model specification | Adjusted R^2^ | *p*-values for coefficients | | |
|  |  | Largest progenitor CTG18.1 allele size | Subject sampling age | Interaction |
| Largest progenitor CTG18.1 allele size only | 76.15% | <0.0001 | NA | NA |
| Subject sampling age only | 0% | NA | 0.59 | NA |
| Largest progenitor CTG18.1 allele and subject sampling age  (no interaction) | 79.84% | <0.0001 | 0.10 | NA |
| Largest progenitor CTG18.1 allele and subject sampling age (with interaction) | 91.02% | <0.0001 | 0.02 | 0.02 |

**Table S3:** Simple and multiple linear regression models using the log-transformed outcome to model the effect of mean progenitor allele size (determined by STR-analysis of leukocyte-derived DNA) and/or subject sampling age per patient on the mean molecule size measured by optical genome mapping in leukocyte-derived DNA per patient. NA: not applicable.
